# Hypermethylation of the enolase gene (*ENO2*) in autism

**DOI:** 10.1007/s00431-014-2311-9

**Published:** 2014-04-17

**Authors:** Yu Wang, Yudan Fang, Fengling Zhang, Miao Xu, Jingzhi Zhang, Jingbin Yan, Weina Ju, W. Ted Brown, Nanbert Zhong

**Affiliations:** 1 Institute of Medical Genetics, Shanghai Children’s Hospital, Shanghai Jiaotong University, Shanghai, China; 2 Institute of Children Health Care, Shanghai Children’s Hospital, Shanghai Jiaotong University, 1440 Beijing West Road, Shanghai, 200040 China; 3 Key Laboratory of Embryo Molecular Biology, Ministry of Health, Shanghai, China; 4 Shanghai Laboratory of Embryo and Reproduction Engineering, Shanghai, China; 5 Peking University Center of Medical Genetics, Beijing, China; 6 New York State Institute for Basic Research in Developmental Disabilities, Staten Island, New York, NY USA

**Keywords:** Autism, Neurodevelopment, Gene *ENO2*, Methylation, Epigenetics

## Abstract

It has been hypothesized that dysregulation of brain-expressed genes is the major predisposing underlying mechanism for autism. This dysregulation may be mediated by differential methylation of CpG sites within gene promoters, which could be candidate biomarkers and used for early clinical screening of autism. A total of 131 pairs of age- and sex-matched autistic and control subjects were recruited in this study. Peripheral blood cells were analyzed. The first five pairs were randomly applied to array-based genome-wide methylation studies. A neuron-specific gene, *ENO2*, was found to be hypermethylated in the autistic samples. This difference was validated by bisulfite sequencing PCR (BSP). The differential expression of *ENO2* gene was further analyzed with RT-qPCR and ELISA. The hypermethylation of *ENO2* within the promoter region was confirmed by BSP to be present in 14.5 % (19/131) of the total of the autistic samples. The mean *ENO2* RNA level in these 19 autistic samples was reduced by about 70 % relative to that in controls. The average level of ENO2 protein expression in the 19 autistic samples (15.18 ± 3.51 μg/l) was about half of that in the controls (33.86 ± 8.16 μg/l). *Conclusion*: These findings suggest that reduced *ENO2* expression may be a biomarker for a subset of autistic children.

**Electronic supplementary material:**

The online version of this article (doi:10.1007/s00431-014-2311-9) contains supplementary material, which is available to authorized users.

## Introduction

Autism comprises a clinically and genetically heterogeneous group of disorders with a complex etiology. It is characterized by impairments in reciprocal social communication and the presence of restricted, repetitive, and stereotyped patterns of behavior [5, 31]. It has an early age of onset, typically before three, and prevalence as high as 1.1 % in the USA [9]. Autism occurs predominantly in males, with a ratio of males to females of about 4 to 1. It is a leading cause of childhood disability and inflicts serious suffering and financial burdens for the family and the society [4].

Autism has been determined to associate with genetic deficiencies. Mutations in several genes, such as *SHANK3*, *NRXN1*, *NLGN3*, *NLGN4*, and *CNTNAPs*, encoding synaptic proteins have been identified to be involved in causing this disease. Studies have been carried out to identify candidate biomarker(s) associated with the development of autism. However, no reliable biomarker to date has yet been found. Altered immune function and complement system deficiency have been reported for some children with autism along with a recurrent incidence of immunological disease [24]. By a proteomic approach, Momeni et al. [14] identified differentially expressed peptides that were fragments of the C3 complement protein and could potentially serve as a set of biomarkers for the early detection of autism. Recent studies [30] found that differential expression of various synaptic genes may affect pathways that are responsible for normal brain development. The differential expression of some of these genes may result from epigenetic modifications [18].

Autism is largely genetic in origin; however, environmental factors may play an important role in autism development. Individual development, especially in the early stages of embryonic development, maternal depression, malnutrition, and contact of certain chemicals or drugs, can increase the susceptibility of autism [24].

Epigenetics may integrate both genetic and environmental influences to regulate neurodevelopmental processes in the etiology of autism [21]. Several genetic syndromes share features with autism, such as Rett, Fragile X, Prader-Willi, and Angelman, and demonstrate dysregulation of epigenetic marks or epigenetic mechanisms [5] (Epigenetic refers to the heritable regulation of various genetic functions [7]). DNA methylation is one of the most extensively studied epigenetic markers. It modulates genomic functions including the regulation of gene differential expression, cellular differentiation, embryonic development, and genomic imprinting [28]. Due to its molecular stability, DNA methylation is considered a key signature of epigenetic changes, and it can be monitored at a genome-wide level.

Recently, researchers found that the methylation profiling of DNA extracted from brain tissues of patients with schizophrenia and bipolar disorder had significant changes comparing to normal controls. These changes included hypermethylation regions of suspected gene loci that had formerly been identified as having a relationship to autism [13]. Interest in the methyl CpG-binding protein 2 (*MECP2*) as a global epigenetic regulator has led to the discovery of increased methylation of the *MECP2* promoter in the frontal cortex of autistic brains [15]. Certain *MECP2* polymorphisms were associated with an increased risk of autism [16]. Methylation profiling of lymphoblastoid cell lines from three sets of identical twins discordant for autism revealed epigenetic differences and led to the identification of a novel autism candidate gene, *RORA*, whose protein product was reduced in autistic brain samples [17]. Collectively, these studies provide support for the potential role of DNA methylation in autism. Most studies of methylation in autism have used brain tissues, but brain tissue cannot be used for early biomarker detection. Therefore, we undertook to study the methylation profile of peripheral lymphocytes from autistic children and their age-matched controls employing a methylation microchip approach. We have focused on the hypermethylation and the differential expression of gene *ENO2*.

## Patients and methods

### Study population

A total of 131 pairs of children of age 3 to 12, autism vs. gender-/age-matched control, were recruited for this study with a protocol that has been approved by the Ethical Committee of the Shanghai Children’s Hospital. Written informed consent was obtained from the parents. All subjects were of Chinese Han ethnicity. The Children fulfilled the diagnostic criteria of autism as defined by the Diagnostic and Statistical Manual of Mental Disorders-IV (DSM-IV) of the American Psychiatric Association. The diagnosis of autism was made based on medical records and interviews by senior psychiatrists with a consensus obtained using the Mini International Neuropsychiatric Interview (MINI) instrument [23]. There were no genetic abnormalities identified in any patients recruited in this study. The control group was recruited from individuals who received regular developmental check-ups in the Outpatient Children Healthcare Clinic of Shanghai Children’s Hospital whose mental status and history of mental illness were evaluated by a senior psychiatrist using the instrument MINI [23]. Detailed information of the participants is provided in Table 1.

**Table 1:** Pairs of subjects recruited in the studies

	Pairs of subjects			Age (years)					
	Total	Male	Female	∼3	∼4	∼5	∼6	∼7	∼12
MeDIP chip study and validation	5	4	1				2	2	1
Additional BSP of *ENO2*	126	96	30	9	28	39	17	16	17
RT-qPCR of *ENO2*	19	14	5	2	2	3	5	3	4
ELISA analysis of ENO2	19	14	5	2	2	3	5	3	4

### Preparation of plasma, DNA, RNA, and cDNA

Venous blood was collected into 3-ml EDTA anticoagulant tubes (Vacutainer System; Becton-Dickinson, Plymouth, UK), and plasma was separated immediately by centrifugation at 1,300*g* for 10 min at 4 °C, and then stored at −80 °C. Genomic DNA was isolated from peripheral leukocytes using a QIAmp DNA Micro Kit (Qiagen, Darmstadt, Germany), following the manufacturer’s instructions. Total RNA was isolated from fresh peripheral blood leukocytes using TRIzol (Invitrogen, Carlsbad, CA), according to the manufacturer’s instructions. DNA and RNA concentrations were measured with a NanoDrop ND-1000 Spectrometer (Thermo Scientific, Boston, MA). Complementary DNA (cDNA) was prepared using Reverse Transcriptase M-MLV (RNase H; Takara, Shiga, Japan).

### Methylated DNA immunoprecipitation (MeDIP) array-based hybridization

In order to screen genome-wide methylation variation, MeDIP array-based hybridization was carried out. Five pairs of subjects from 131 pairs of autism-control cases were subjected to MeDIP study (Table 1). Five micrograms of genomic DNA (gDNA) was sheared to an average length of 200–500 bp with ultrasound. Autistic gDNA was incubated with 5 μg of anti-5-methyl cytidine antibody (Epigentek, New York, NY) but the control gDNA was incubated with 5 μg of IgG. The MeDIP-enriched and input genomic DNA samples were labeled with Cy5 and Cy3, respectively, and hybridized to a NimbleGen HG18 CpG Promoter array (Roche NimbleGen, Madison, WI). MeDIP is a method of high throughput screening of gene methylation differences. In our study, an antibody specific for methylated cytosine was used to capture methylated genomic fragments, which were hybridized against NimbleGen oligonucleotide CpG promoter array that includes 15,936 UCSC-annotated CpG islands and 17,354 RefSeq gene promoter regions (covering approximately −1,300 to +500 bp from the transcription start site). Scanning was performed with an Axon GenePix 4000B microarray scanner (Molecular Devices, Sunnyvale, CA).

### Bisulfite sequencing PCR (BSP)

In order to confirm the different methylation status of *ENO2* gene identified by MeDIP, bisulfite treatment was performed using a DNA Methylation Kit (GMS, Reston, VA), following the manufacturer’s instructions. Bisulfite-treated DNA was stored at −80 °C until needed.

BSP primers were designed with an online program MethPrimer (http://www.urogene.org/meth-primer/index1.html) for Primer F (5′-TGTGAGATATTGAGGTTGTTTTG-3′) and primer R (5′-ACCCCCRATAAATCAAAAACTA-3′) to amplify an amplicon with 252 bp, which corresponds to the region detected by the microarray. The sequence of primers was blasted against a gene bank in NCBI. In total, 1 μl bisulfite-modified DNA (50 ng/μl), 2 μl dNTPs (2.5 mM each), 1 μl of each forward (F) and reverse (R) primer (10 μM), 1.5 μl MgCl2 (25 mM), 2.5 μl 10 × PCR buffer, and 0.25 μl AmpliTaq polymerase (5 U/μl; Applied Biosystems, Foster City, CA) were combined in a final volume of 25 μl. Reactions were incubated for 2 min at 94 °C and then for 35 cycles at 94 °C denaturing for 20 s, 58 °C annealing for 30 s, and 72 °C extension for 30 s followed by a final 7 min extension at 72 °C. To confirm amplification of the 252 bp product, 10 μl of the completed PCR product was resolved using a 3 % agarose gel in 1 × Tris-borate EDTA and visualized with ethidium bromide. Bisulfite sequencing PCR products were sequenced with a DNA analyzer (ABI 3730, Long Beach, CA) after being cloned into a pMD18-T-vector. For each sample, 10 colonies were sequenced.

### RT-qPCR

In order to analyze the expression level of *ENO2* gene, one microgram of total RNA and 20 pmol of real time PCR downstream primers were used in a 20-μl reverse transcription reaction with a protocol supplied with a Reverse Transcriptase M-MLV (RNase H) kit. Quantitative Real Time PCR (RT-qPCR) was performed on the above RT products. The sequences of primers and probes were as follows: *ENO2*-F (5′-GGGAACTCAGACCTCATCCTG-3′), *ENO2*-R (5′-CTTGTTGCCAGCATGAGAGC-3′), *ENO2*-Probe (5′-FAM-TGTGCCGGCTTCAACGGATC-TAMRA-3′), β-actin-F (5′-CCTGGCACCCAGCACAAT-3′), β-actin-R (5′-GCTGATCCACATCTGCTGGAA-3′), and β-actin-Probe (5′-FAM-ATCAAGATCATTGCTCCTCCTGAGCGC-TAMRA-3′). In total, 2 μl of each forward (F) and reverse (R) primer (10 μM), 2 μl MgCl2 (25 mM), 2.5 μl 10 × *Ex Taq* buffer (Mg^2+^-free), probe (6.25 μM), 2 μl dNTPs (2.5 mM each), and 0.25 μl *Ex Taq* polymerase (5 U/μl; Takara, Shiga, Japan) were used for a total of 25 μl of solution for the PCR reactions. The PCR program started at 95 °C for 5 min followed by 40 cycles of 95 °C for 30 s and 59 °C for 30 s. Fluorescent Amplisensor marker (FAM) at the wavelength of 510 nm was collected at 59 °C. The signal was analyzed by software 2.0.1 installed on the real time PCR apparatus (ABI 7500, Long Beach, CA). The amplified β-actin gene was used as an internal control.

### Enzyme-linked immunosorbent assay (ELISA)

In order to analyze the expression level of ENO2 protein in plasma, an ELISA kit (R&D system, Minneapolis, MN) was used according to the manufacture’s protocol. The optical density of each well was determined within 30 min with a microplate reader BioTek synergy 2 (BioTek, Shanghai, China), which was set to 450 nm.

### Statistical analysis

Statistical analyses were performed using the SPSS software package, version 16.0 (SPSS Inc, Chicago, IL). For microarray data analysis, raw data were extracted as pair files with NimbleScan^TM^ software. We performed the raw data normalization. The ratio of the methylated to the input signal for each sequence spotted on the array was adjusted, calculated, and used for read-out of methylation level by the software. Detection of *peaks* with a series of default procedures-specified parameters was performed to ensure that the differences were statistically significant (*p* < 0.01). The differentially methylated genes between the two groups were defined as at least a 1.5-fold change of geometric mean intensity; Student’s *t* test was used for methylation percentage and messenger RNA (mRNA) and protein expression level expression. The standard deviation (SD) was calculated. A two-tailed *p* value of less than 0.05 was considered statistically significant.

## Results

### Genome-wide DNA methylation pattern of autism in peripheral blood

**Fig. 1:**
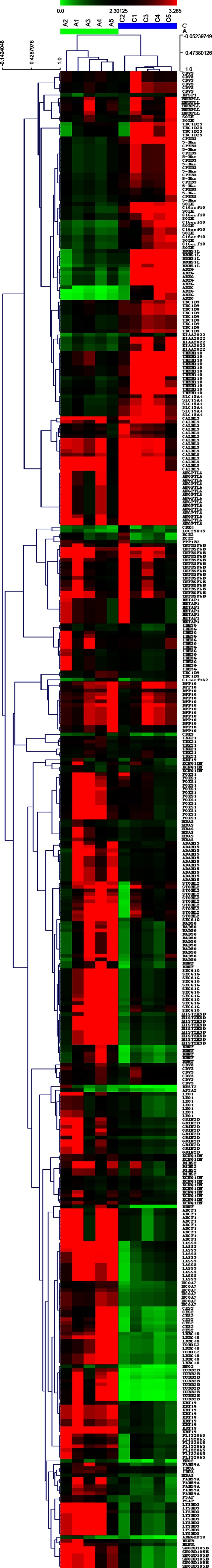
Microarray assay (MeDIP) of genome-wide differential methylation among five pairs of autistic (*A*) and control (*C*) DNAs. Cutoff of cluster aggregation of differentially methylated genes was pre-set as 1.5-fold, *p* < 0.05, and detected by MeDIP in ≥3 pairs of samples. Fifty-one genes were shown upregulated (*red*) and 46 downregulated (*green*)

**Fig. 2:**
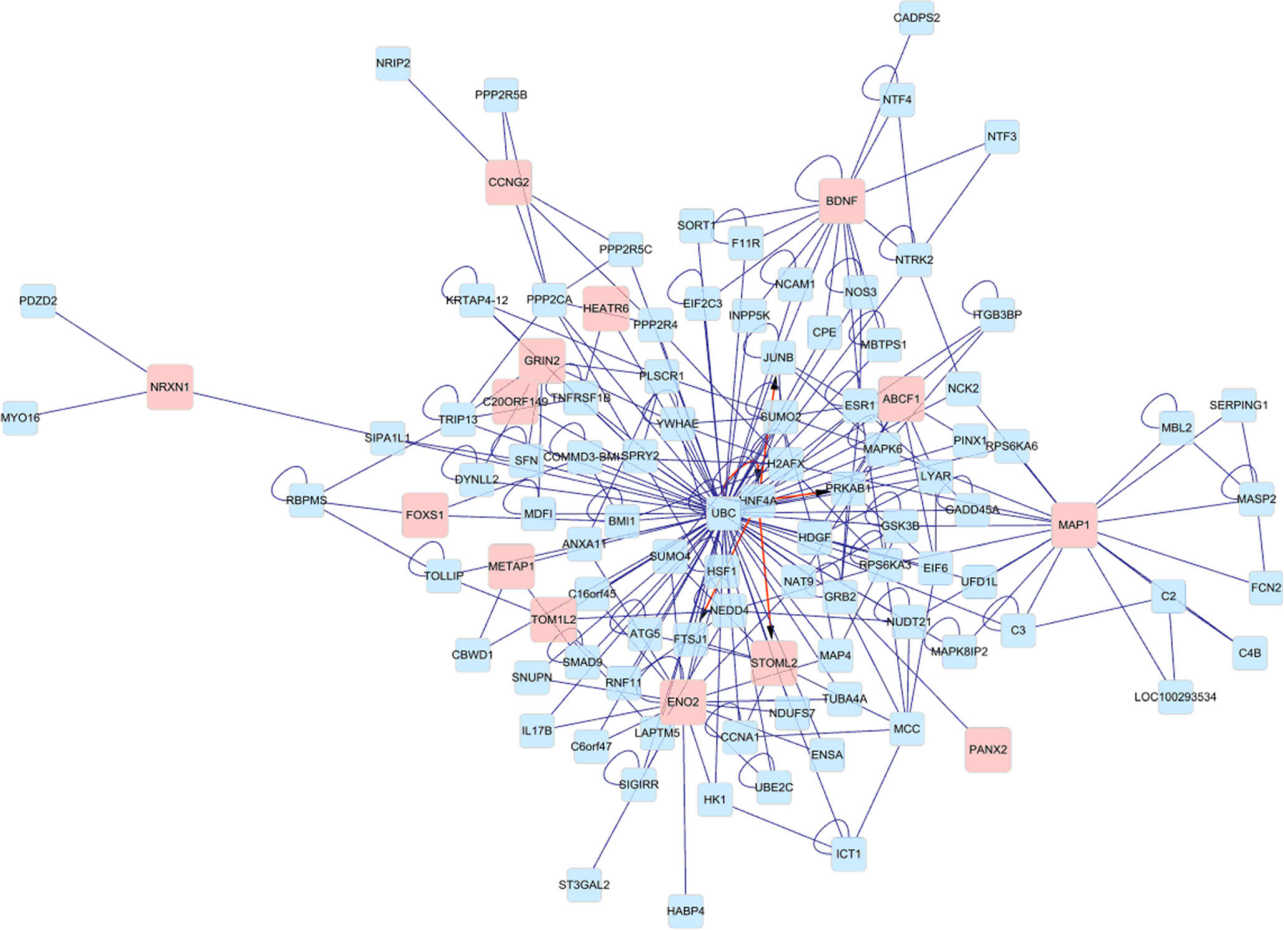
Network analysis was performed to examine the relationship between the synaptic regulation set of genes and biological processes. Protein-protein interactions are labeled with *blue* and protein-DNA interactions with *pink*

There were 228 genes differentially methylated in the promoter regions and 247 genes in CpG islands in the five pairs analyzed by the MeDIP method when 1.5-fold and *p* < 0.05 were pre-set as the cutoff criteria. Compared to the control group, 7 genes were found to be hypermethylated in the autistic group from all five pairs. In addition, 19 were hypermethylated among four pairs and 23 among three pairs. Compared to the control group, 6 genes were found to be hypomethylated among all five pairs, 30 among four pairs, and 10 among three pairs, as shown in Table 2. These genes were clustered (Fig. 1), and the average linkage clustering was determined to examine the relationship between genes of synaptic regulation and biological processes using the Pearson correlation, as shown in Fig. 2.

**Table 2:** Differential methylation of genes detected from microchip arrays

Autism vs. control	Hypermethylation	*p* value	Hypomethylation	*p* value
5 pairs	*IDUA*	0.0008	*RTN3*	0.0012
	*HEATR6*	0.0009	*SLC15A4*	0.0013
	*LASS3*	0.0009	PODNL1	0.0015
	*TOM1L2*	0.0012	*HECW1*	0.0018
	*LRRC48*	0.0013	*IDH3G*	0.0019
	*SNORD105B*	0.0015	*SPSB4*	0.0021
	*ANGPTL6*	0.0015		
4 pairs (In addition to genes listed in 5 pairs above)	*METAP1*	0.0005	*TMEM110*	0…09
	*MAP1*	0.0005	*PCDH10*	0.0009
	*BOLIG3*	0.0006	*TBC1D9*	0.0012
	*ABCF1*	0.0006	*AREG*	0.0012
	*BEST2*	0.0007	*HES3*	0.0012
	*GRIN2*	0.0009	*PWWP2*	0.0013
	*DKDELR1*	0.0009	*BCHST3*	0.0013
	*FOXS1*	0.0011	*MARCH5*	0.0013
	*C20orf149*	0.0023	*CPEB3*	0.0026
	*ENO2*	0.0024	*IGSF9B*	0.0029
	*PANX2*	0.0025	*AP2A2*	0.0035
	*PEAR1*	0.0026	*F7*	0.0038
	*CCNG2*	0.0031	*C14orf73*	0.0041
	*BDNF*	0.0036	*BRMS1L*	0.0051
	*CES2*	0.0045	*C14orf162*	0.0061
	*FAM96*	0.0051	*BCL11B*	0.0071
	*BNGEF*	0.0062	*SOLH*	0.0082
	*FAM19A5*	0.0068	*C16orf10*	0.0081
	*STOML2*	0.0075	*CDK3*	0.0085
			*GRIN2D*	0.0089
			*ARHGEF18*	0.0091
			*HNRPLL*	0.0093
			*BFSP1*	0.0093
			*C20orf134*	0.0095
			*TNFRSF6B*	0.0096
			*TBC1D23*	0.0096
			*KIAA1257*	0.0097
			*CDV3*	0.0098
			*CXorf38*	0.0099
			*KIAA2022*	0.0099
3 pairs (In addition to genes listed in 5 and 4 pairs above)	*NCOA7*	0.0004	*HIST2H3D*	0.0002
	*TUBB2B*	0.0004	*RORC*	0.0030
	*CALML3*	0.0004	*ADAM15*	0.0004
	*PSAP*	0.0004	*ECE2*	0.0005
	*KCNQ1DN*	0.0011	*PPP1R2*	0.0016
	*HRAS*	0.0015	*LYSMD3*	0.0017
	*CDX2*	0.0016	*RAD50*	0.0021
	*SLC24A2*	0.0018	*PITX1*	0.0029
	*MLNR*	0.0021	*IRX1*	0.0032
	*LEO1*	0.0026	*LOC25845*	0.0043
	*CHRNA3*	0.0029		
	*KRT19*	0.0031		
	*TBX21*	0.0035		
	*FLJ32065*	0.0039		
	*CBX4*,	0.0041		
	*NRXN1*	0.0045		
	*FAM59A*	0.0059		
	*GTPBP3*	0.0067		
	*PNMAL2*	0.0073		
	*DPP10*	0.0075		
	*CYP2D7P1*	0.0081		
	*SEC61G*	0.0086		
	*RIMS2*	0.0087

From Table 2, four genes *LASS3*, *PANX2*, *SLC15A4*, and *ENO2* were selected for further study based on the following concerns: (1) significant difference of methylation status among these genes that were identified with MeDIP array in five pairs of autism-control cases; (2) gene function(s), more importantly, their potential roles that had been reported previously in the development of autism [1, 30]; and (3) their expression in the peripheral blood, which might be a potential candidate of biomarker for assisting clinical recognition and early detection of autism.

### Validation of hypermethylation of *ENO2* gene

**Fig. 3:**
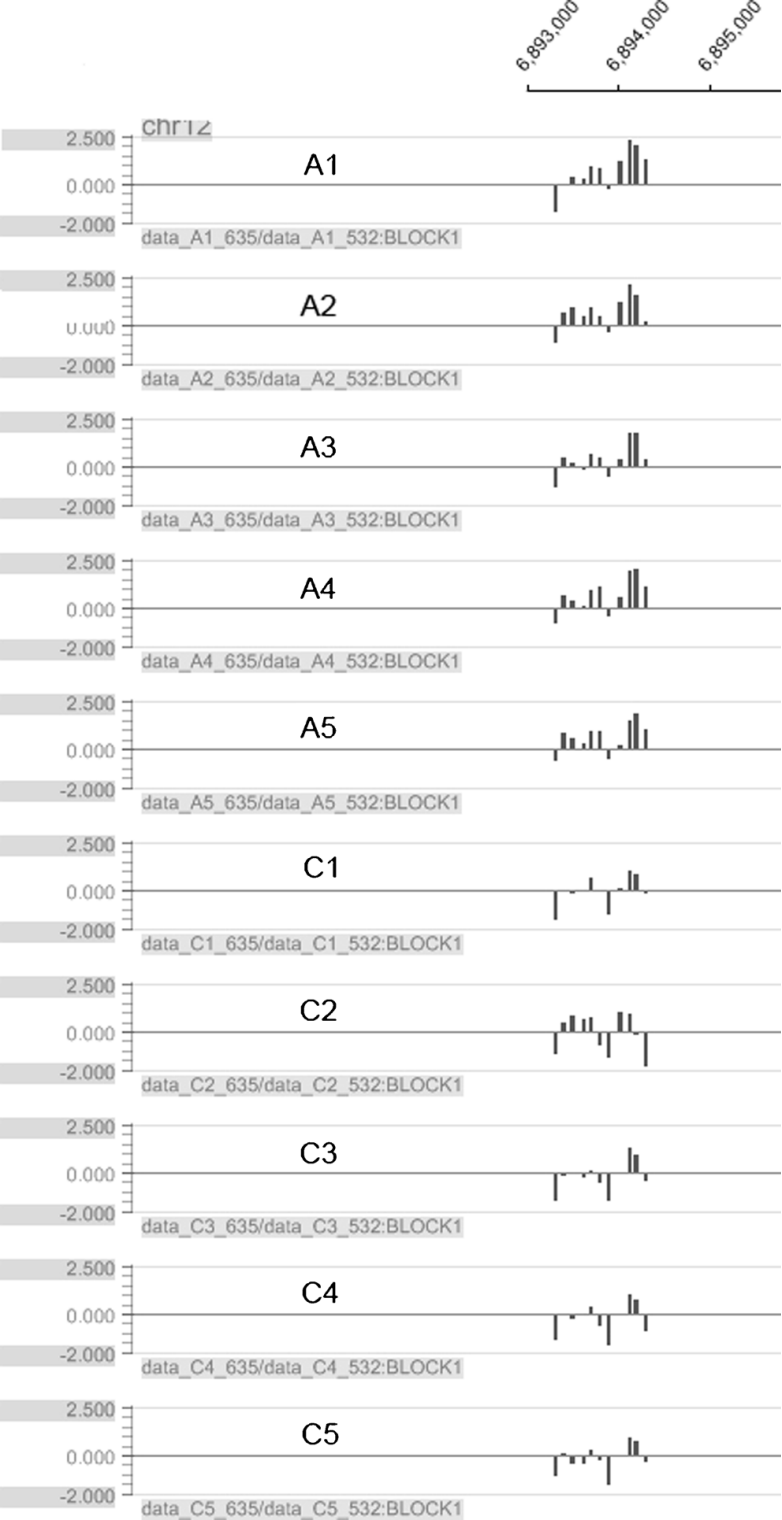
The hypermethylation of the *ENO2* gene in the CpG promoter microarray. Output of differential methylation of *ENO2* gene was obtained from five pairs of autistic (*A*) vs. control (*C*) children. *Numbers* at the top of the graphs are the genomic positions

**Fig. 4:**
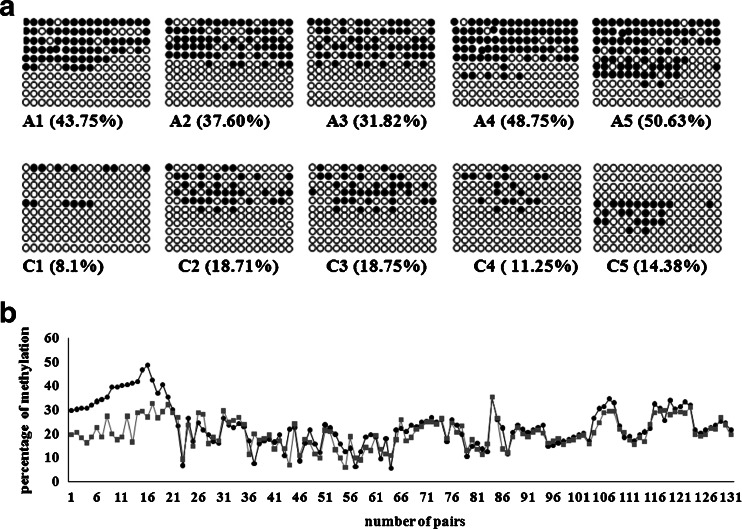
BSP-sequencing analysis confirmed the hypermethylation on these five pairs of DNAs (**a**). The *filled dark circles* denote methylated cytosine, while the *open circles* represent unmethylated cytosine. To plot all, 131 pairs of autism vs. control DNAs (**b**) show that the *gray squares* (autism) are apart from *black circles* (control) for the first set of 19 samples (*X*-axis #1–19) but overlapped among the remaining 112 samples, which gave a total of 14.5 % (19/131) of the autism children with hypermethylation of the *ENO2* gene

The differentially methylated genes *LASS3*, *PANX2*, *SLC15A4*, and *ENO2* were verified by BSP with the five pairs of samples that were used originally for the MeDIP analysis. We found that *SLC15A4*, which was hypomethylated in five out of five pairs by MeDIP, did not reveal hypomethylation by BSP that is likely a disagreement between the MeDIP and BSP, as previously reported [19]. The experimental principles of measuring methylation status with MeDIP array or with BSP are different. MeDIP was a large-scale purification technique, which was used to enrich for methylated DNA sequences. It consists of isolating methylated DNA fragments by an antibody raised against 5-methylcytosine. While in BSP sequencing, cytosine is converted into uracil residue and then recognized as thymine in subsequent PCR amplification. However, 5-methylcytosine is resistant to this conversion and remained as cytosine, allowing methylated cytosine to be distinguished from unmethylated cytosine. Data is calculated according to the average probe signals covering the specific gene in MeDIP array, in which false-positive results might be emerged. Until now, BSP is the gold standard for the detection of single-base methylation. Two genes, *LASS3* that was hypermethylated in five out of five by MeDIP and *PANX2* in four out of five, did not give a satisfactory PCR amplification with a very lowly detectable reverse transcription PCR (RT-PCR) signal, primers are shown in Supplementary Table [1] (online). The genes we selected potentially would be used as the biomarkers for a subset of autistic children. They should be expressed in peripheral blood. Considering the technical performance, RT-PCR would be easier, while BSP is time consuming. Therefore, we selected RT-PCR as our platform for validating the differential methylation in peripheral blood. If the gene had very lowly detectable RT-PCR signals that did not give a satisfactory PCR amplification, we would not go on carrying out BSP. Only the *ENO*2 gene, which was hypermethylated in four out of five pairs, was found to have good PCR amplification, which was consistent with the outcome of the MeDIP (Fig. 3) and that could be subjected to further confirmation by BSP sequencing. By this approach, the autistic group had an average of 42.51 % of CpGs methylated as compared to the control group of 14.24 % (*p* < 0.01; Fig. 3a). Following up on this result, a larger sample size was tested for hypermethylation of *ENO2* that encodes the isozyme enolase-alpha. Ninety-six pairs of autism vs. normal control samples were subjected to quantitative BSP analysis of methylation of the *ENO2* gene promoter region. The original study was performed among 101 pairs (96 pairs in addition to 5 pairs), in which 16 were found to have hypermethylation in *ENO2* promoter region. To further confirm the methylation alteration, an additional set of 30 pairs was analyzed. In this independent confirmation study, 3 out of 30 (10 %) were shown to have hypermethylation, which gave a total number of 19 out of 131 pairs (14.5 %) showing hypermethylation (Fig. 4b). Among the 19 autism-control pairs of cases, the autistic group had an average of 39.61 % of CpGs methylated, while the control group had 18.81 %, showing a significant difference (*p* < 0.01). Within the 19 pairs, 12 were 6 to12 years old, accounting for 9.2 % of the 131 total and 24 % of this age group; 7 were between 3 to 5 years of age, accounting for 5.3 % of the total and 9.2 % of this age group, as shown in Table 1.

### Decreased transcription and translation of *ENO2*

**Fig. 5:**
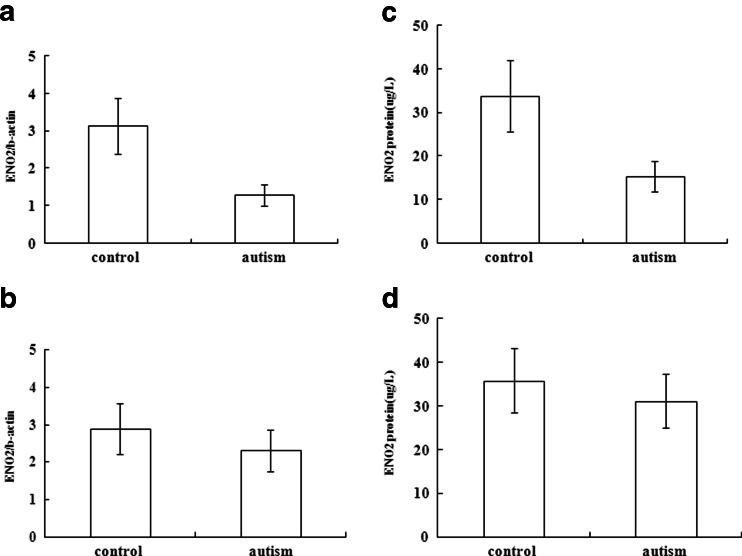
Quantitative analysis of *ENO2* gene expression. Both RT-PCR for mRNA (**a**) and ELISA for protein (**b**) of *ENO2* gene showed significant reduction (*p* < 0.01) among 19 pairs of autistic vs. control children. Both RT-PCR for mRNA (**c**) and ELISA for protein (**d**) of *ENO2* gene showed no significant difference among the remaining 112 pairs of autistic vs. control children

To investigate whether the hypermethylation of *ENO2* found in our autistic samples resulted in a reduction in gene expression, we measured mRNA levels by Quantitative RT-PCR (RT-qPCR) of the *ENO2* gene in peripheral blood samples from the 19 pairs of autism vs. control. As shown in Fig. 5a, the mean mRNA level of the *ENO2* gene in the autistic samples was significantly lower than in controls, with a 70 % reduction of *ENO2* mRNA (*p* < 0.01). ELISA analysis was used to determine the translational level of the ENO2 protein in these 19 pairs of plasma samples (Fig. 4b). The average level of ENO2 protein expression in the autistic samples (15.18 ± 3.51 μg/l) was about half of that in controls (33.86 ± 8.16 μg/l; *p* < 0.01). However, among 112 pairs that did not show the hypermethylation difference, there was no decreased expression of mRNA or ENO2 protein (*p* > 0.05; Fig. 5c, d).

### The relation between the hypermethylation of *ENO2* gene and environmental factors

The environmental factors including parent, child, and family characteristics were presented in Table 3. Correlation between the hypermethylation of *ENO2* gene and environmental factors was further analyzed. We found that compared to the non-hypermethylation group, the hypermethylation group had a lower Apgar score at 5 min. In addition, length of breast feeding and daily TV time both had significant difference between two groups (Table 4).

**Table 3:** Information of autistic children and their parents

Characteristics	Value (%)		*p* value
	Autism	Control	
Maternal characteristics			
Age at pregnancy (years)			
<30	51.5	65.4	0.05
30–35	37.6	29.7	
>35	10.9	4.9	
Occupation (%)			
Housewife	58.4	50.2	0.12
Working mother	41.6	49.8	
Education (%)			
Tertiary education	36.7	38.2	0.35
Secondary school	39.2	34.3	
Primary school	24.1	27.5	
Medical history			
Caught a cold during the 1st trimester	16.8	14.9	
Mental illness	4.9	0	
Paternal characteristics			
Smoking (%)	60.2	62.1	0.25
Alcohol consumption (%)	74.5	70.1	
Mental illness (%)	1.8	0.9	
Family characteristics			
Monthly income (%)			
>10,000	30.5	34.2	0.08
5,000–10,000	36.8	35.3	
3,000–<5,000	22.1	21.0	
<3,000	10.6	9.5	
Child characteristics			
Gestational weeks (%)			
<37	21.8	11.9	0.04
37–40	68.3	80.2	
>40	9.9	7.9	
Apgar score at 5 min (%)			
8–10	83.2	96.5	0.03
4–7	16.8	3.5	
Length of breast feeding			
<4 months	21.1	19.7	0.06
4–6 months	42.3	38.9	
>6 months	36.6	41.4	
Child raised by			
Parents	12.9	20.8	0.03
Grandparents	59.4	49.5	
Nanny	27.7	29.7	
Daily TV time			
<1 h	39.6	50.5	0.04
1–2 h	32.7	29.7	
>2 h	27.7	19.8	
Medication (%)			
Ritalin	20.8	0	0.04
Risperdal	7.9	0	
No specific medication	71.3	100

**Table 4:** Different factors related to the methylation of *ENO2* gene between the hypermethylation and non-hypermethylation groups ($\overline{\mathrm{X}}$ ± SD)

	Hypermethylation (*n* = 19)	Non-hypermethylation (*n* = 112)	*t*	*p*
Length of breast-fed (months)	4.72 ± 1.19	7.21 ± 1.91	2.539	0.019
Apgar score at 5 min	7.12 ± 2.19	9.21 ± 1.83	2.419	0.026
Daily TV time (h)	2.06 ± 0.82	1.15 ± 0.35	2.149	0.032

## Discussion

The number of published studies comparing differences in blood DNA methylation between cases and controls for complex diseases is increasing. Toperoff et al. found a specific methylation pattern in whole blood from patients with type 2 diabetes that could be detected prior to the onset of the disease [29]. It is important to note that cell heterogeneity may act as a confounder when measuring DNA methylation in whole blood, and the possibility to adjust for differential cell counts is being investigated [29]. Differences in DNA methylation were identified in the leukocytes of mothers of children with congenital heart defects [2]. In a recent study, adults with Asperger syndrome were shown to exhibit a sex-specific expression of serum biomarkers [22]. A proteomic study on autopsied autism brains using 2-D gel electrophoresis revealed a single-nucleotide polymorphism in glyoxalase [8]. A proteomic study of serum from children with autism also showed a differential expression of apolipoproteins and components of complement [3]. All these studies leave an open question of whether the differentially expressed proteins are the result of altered epigenetic methylation.

Our result has demonstrated that hypermethylation of a single gene, *ENO2*, may be associated with about 15 % of autistic cases. Recent studies have identified *ENO2* as a gene involved in the development of autism [30]; differential expression of the *ENO2* gene in the Wernicke area in patients with schizophrenia [11] and in the anterior cingulated cortex in female schizophrenia patients [12]. Lastly, a genetic study reported that several SNP haplotypes of the *ENO2* were associated with schizophrenia [8], suggesting that *ENO2* may be involved in the early life. Although it is not definitively sure at present whether hypermethylation of *ENO2* gene is a specific factor to autism and if this epigenetic abnormality is a part of the epigenetic mechanisms that may result in autism, or if it is a consequence from other etiological factors, our finding has opened a new avenue to further investigate the epigenetic pathogenesis for autism. Although it is not possible to study directly brain tissues of these patients, the study of other available tissues would add some clues to support this hypothesis. Induced pluripotent stem (iPS) cells derived from autistic patients are being under process to be differentiated to neuron, which will help to study the methylation status.

The MeDIP results showed that four out of five pairs of autistic children, 6 to 12 years of age, showed differential hypermethylation of *ENO2*. Among 126 additional pairs analyzed with BSP, 15 cases had differential hypermethylation. Looking into the details on the overall age range of this group of 101 children, we noticed that 76 (60 %) had an age of 3 to 5, and 50 (40 %) were 6 to 12 years old. The percentage hypermethylation of ENO2 among the younger autistic children (7/76 = 9.2 %) was lower than that in the older autistic children (12/50 = 24 %). Two possible reasons can be suggested for this difference: either our sample size was too small, due to autism being a highly heterogeneous complex disease, with many genes/loci and genetic variations involved, rather than being a distinct neurodevelopmental disorder [1, 31], or the methylation of *ENO2* gene may have a “time course,” which could be in agreement with the DNA methylation process associated with brain development [6]. All 19 children from the group with *ENO2* hypermethylation have significant language development disorder. They almost have no language spoken and could not use language to communicate, while the other 112 children had more or less spoken language. Accordingly, we hypothesize that *ENO2* may have a function in language development.

It has been noticed that the initial methylation study involved five pairs of subjects. The hypermethylation of *ENO2* was identified in four out of five pairs (80 %). In the larger sample studied the proportion of patients with hypermethylated *ENO2* that was found to be 14 %. Thus, there is a very significant difference between the pilot study and the larger study in terms of proportion of abnormal methylation. Even after correction for age, the proportion remains very significantly different (24 % in older pairs with autistic children vs. controls). To address this discrepancy, our explanation is that autism is a highly heterogeneous neurodevelopmental disorder with a complex genetic etiology and phenotype(s) overlapped with some monogenic genetic diseases, such as the fragile X syndrome [1], which made the clinical-diagnosed “autism” as a spectrum of disorders, rather than a distinct clinical disease. This means that the symptoms of autism can be present in a variety of combinations with a range of severity. The disease has variable cognitive manifestations, ranging from a non-verbal child with mental retardation to a high-functioning college student with above-average IQ with inadequate social skills. Therefore, it is not surprising to us to see the discrepancy from clinical point of view. In our study, the initial five cases subjected to the discovery study with MeDIP were well characterized as they all have a similar and typical autistic clinical feature with the characteristic communication disabilities. However, the remaining larger set of the cases for confirmation was more heterogeneous. From the technical point of view, RT-PCR experiments were carried out manually, but MeDIP was done automatically, which could be another factor influencing the results. Further confirmation with well-diagnosed and well-characterized larger size of autism cases would be necessary to assure that hypermethylation of *ENO2* could be applied as a clinical biomarker for early detection of autism.

Earlier study on genome-wide methylation identified that *RORA* gene was abnormally methylated that resulted in decreased expression of RORA protein in peripheral blood [17]. However, our data did not show a replicated result that *RORA* gene is hypermethylated at least not in three out of five and above. Similarly to the above discussion, we believe this is due to the heterogeneity and the difference of technique platform.

Elevated plasma levels of ENO2 have been found to be associated with traumatic brain injury [20] and lung cancer [26]. Emerging evidence also suggests that *ENO2* is associated with psychiatric disorders. Liao et al. [10] detected 924 differentially expressed gene transcripts using a total gene expression microarray in lymphoblastoid cell lines between male heroin-dependent individuals and control subjects, and verified the reduced expression of *ENO2* in heroin-dependent individuals using RT-qPCR and Western blot analysis. It was reported that reduced expression of *ENO2* in heroin-dependent individuals may confer increased risk for heroin dependence. Autoantibodies against an epitope conserved in three isoforms of enolase were detected in the sera of patients with schizophrenia [25]. Elevated neuronal enolase in the nucleus accumbency of cocaine overdose victims was also reported [28]. Moreover, increased *ENO2* expression was detected in the Wernicke area in the postmortem brains of subjects with schizophrenia [11].

## Conclusion

Our findings indicate that downregulation of *ENO2* may be associated with a subset of autism; however, it remains an open question of whether the *ENO2* gene is a key component in brain development, and if it is differentially expressed in the embryo or fetal tissues at early stages of gestation. The reduced *ENO2* expression may be a biomarker for a subset of autistic children.

## Electronic supplementary material

Below is the link to the electronic supplementary material.

Table S1 (DOCX 13 kb)

## Abbreviations

BSP: Bisulfite DNA sequence PCR
DSM-IV: Diagnostic and Statistical Manual of Mental Disorders-IV
ELISA: Enzyme-linked immunosorbent assay
MECP: Methylation-combined protein
MeDIP: Methylated DNA immunoprecipitation
MINI: Mini International Neuropsychiatric Interview
PCR: Polymerase chain reaction
RT-PCR: Reverse transcription PCR
