# Supplementary material for: Hypermethylation of the enolase gene (ENO2) in autism
Source: Eur J Pediatr. 2014 Apr 17;173(9):1233–44. doi: 10.1007/s00431-014-2311-9 (PMC4134484; doi:10.1007/s00431-014-2311-9)
Supplement: Supplementary file 1 — (DOCX 13 kb) [file 431_2014_2311_MOESM1_ESM.docx]

Table S1 Primers of *LASS3*、*PANX2* and *SLC15A4*

| Gene name | Primer-F | Product(bp) |
| --- | --- | --- |
| *LASS3* | F: 5'-TGATCTTGCAGGTCCTTCAC-3' | 448 |
|  | R: 5'-TGACCAGTCTGAGTCCTAAC-3' |  |
| *PANX2* | F: 5'-AATGTCTGTCAGGCGACAAG-3' | 347 |
|  | R: 5'-CTGCACATCAACACGCTATC-3' |  |
| *SLC15A4* | F: 5'-AGTTGCTCTGTGGCTTGAGATG-3' | 623 |
|  | R: 5'-GACTGCTGGCACTGGTGTCTAT-3' |  |
